# Decrypting the Possible Mechanistic Role of Fenofibrate in Alzheimer's Disease and Type 2 Diabetes: The Truth and Mystery

**DOI:** 10.1111/jcmm.70378

**Published:** 2025-03-04

**Authors:** Mansour A. Alsaleem, Hayder M. Al‐Kuraishy, Ali I. Al‐Gareeb, Maha M. Abdel‐Fattah, Mohammed Alrouji, Nasser A. Al‐Harchan, Mubarak Alruwaili, Marios Papadakis, Athanasios Alexiou, Gaber El‐Saber Batiha

**Affiliations:** ^1^ Unit of Scientific Research, Applied College Qassim University Buraydah Saudi Arabia; ^2^ Department of Clinical Pharmacology and Medicine, College of Medicine Mustansiriyah University Baghdad Iraq; ^3^ Department of Clinical Pharmacology Jabir Ibn Hayyan Medical University Kufa Iraq; ^4^ Department of Pharmacology and Toxicology, Faculty of Pharmacy Beni‐Suef University Beni‐Suef Egypt; ^5^ Department of Clinical Laboratory Sciences, College of Applied Medical Sciences Shaqra University Shaqra Saudi Arabia; ^6^ Department of Clinical Pharmacology, College of Dentistry Al‐Rasheed University Baghdad Iraq; ^7^ Department of Internal Medicine, College of Medicine Jouf University Sakaka Saudi Arabia; ^8^ University Hospital Witten‐Herdecke University of Witten‐Herdecke Wuppertal Germany; ^9^ University Centre for Research & Development Chandigarh University Mohali India; ^10^ Department of Science and Engineering Novel Global Community Educational Foundation Sydney New South Wales Australia; ^11^ Department of Research & Development Funogen Athens Greece; ^12^ Department of Pharmacology and Therapeutics, Faculty of Veterinary Medicine Damanhour University Damanhour, AlBeheira Egypt

**Keywords:** Alzheimer's disease, fenofibrate, T2D

## Abstract

Alzheimer's disease (AD) is a neurodegenerative disease caused by the progressive deposition of extracellular amyloid beta (Aβ) and intracellular neurofibrillary tangles (NFTs). Of note, metabolic disorders such as insulin resistance (IR) and type 2 diabetes (T2D) are associated with the development of brain IR and associated neurodegeneration. In addition, AD neuropathology and linked cognitive impairment accelerate the development of peripheral IR and the progression of T2D. Therefore, there is a bidirectional relationship between T2D and AD. It has been demonstrated that AD and T2D induce dysregulation of peroxisome proliferator‐activated receptor alpha (PPAR‐α) leading to the central and peripheral metabolic disturbances. Hence, dysregulated PPAR‐α could be a shared mechanism in both AD and T2D, and restoration of PPAR‐α signalling by PPAR‐α agonist fenofibrate (FN) may alleviate T2D and AD. Therefore, this review aims to shed light on the potential involvement of PPAR‐α in T2D and AD, and how FN could be effective in the management of AD. FN seems to be effective in both AD and T2D by dual neuroprotective and antidiabetic effects that can mitigate AD neuropathology and T2D‐related complications by modulating various cellular processes and inflammatory signalling pathways. In conclusion, FN could be a possible candidate in the management of AD and T2D by modulating different signalling pathways involved in the pathogenesis of these conditions.

## 1 Introduction

**FIGURE 1:**
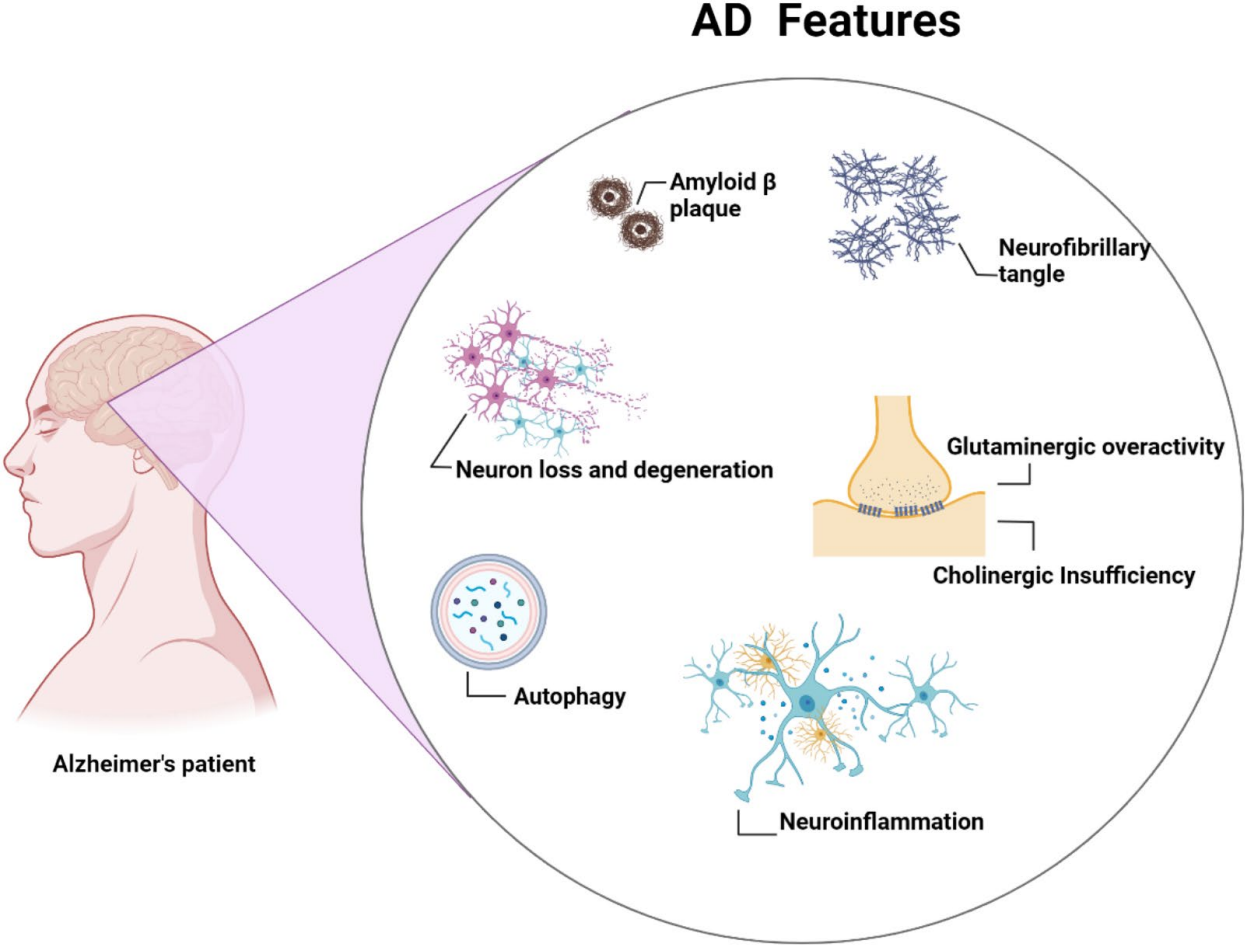
Pathophysiology of AD: Progressive accumulation of the extracellular amyloid beta (Aβ) and intracellular neurofibrillary tangles (NFTs) induce neurodegeneration with subsequent cholinergic dysfunction and glutamatergic overactivity. These neuropathological changes also inhibit neuronal autophagy and trigger the development of neuroinflammation.

Alzheimer's disease (AD) is a neurodegenerative disease caused by the progressive deposition of extracellular amyloid beta (Aβ) and intracellular neurofibrillary tangles (NFTs) [1, 2]. Multiple processes, including oxidative stress, autophagy, inflammation, cholinergic dysfunction and mitochondrial dysfunctions are involved in AD neuropathology [3, 4] (Figure 1).

It has been revealed that Aβ plaques are crucial in the development of AD [5]. When plaque sequestration ability is diminished, soluble Aβ can spread into extracellular causing severe synaptic dysfunction and neuronal injury [5]. Similarly, Aβ oligomers affect cell membrane ion channels and receptors to activate both intracellular and extracellular neurotoxicity [5, 6]. These pathological changes stimulate an extreme imbalance between inhibitory and excitatory neurotransmitters with the progress of hyperexcitability [6]. It has been shown that imbalance of the excitatory/inhibitory axis was correlated with the severity of Aβ deposition in AD patients compared to healthy controls [7]. Therefore, Aβ‐induced neuronal damage and the progression of increased AD are facilitated by synaptic dysfunction and the increase of excitatory/inhibitory imbalance. Importantly, AD represents 70% of all dementia types, and the majority of AD cases are late‐onset AD, meaning they develop sporadically beyond the age of 65 [8]. However, 5% of AD is of genetic type caused by mutations in the amyloid precursor protein (*APP*) genes and presented as an early‐onset AD [9, 10].

**FIGURE 2:**
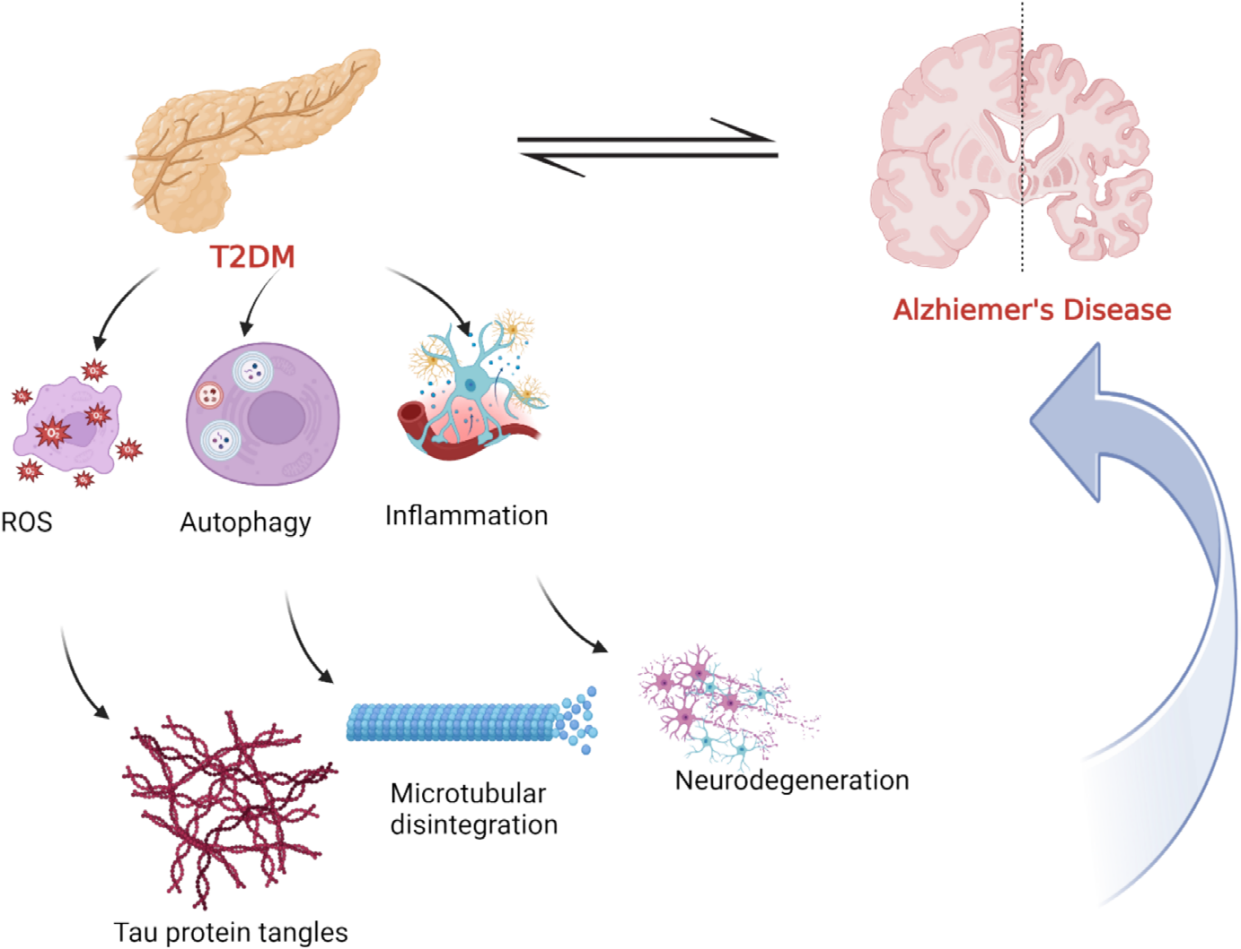
The relationship between AD risk and T2D: T2D inhibits autophagy, and activates the release of reactive oxygen species (ROS) and pro‐inflammatory cytokines leading to the formation of tau protein tangles and microtubular disintegration as well as neurodegeneration.

Mounting evidence supports the link between type 2 diabetes (T2D) and the onset of AD [10, 11]. Notably, brain insulin resistance (IR) and inhibition of neuronal insulin receptors are commonly associated with the advancement of T2D [11]. These changes reduce brain glucose metabolism, increase Aβ_1‐42_ production, impair Aβ clearance and trigger the deposition of aberrant Aβ plaque [12, 13]. Accordingly, it has been reported that T2D patients have a 50%–75% chance for developing AD, while people with AD have a greater chance for developing T2D [14] (Figure 2). According to the in vitro study, high blood glucose level causes apoptosis in rat embryonic cortical neurons. High blood glucose level induces the phosphorylation of tau protein and the formation of Aβ [14]. In addition, a study conducted on mice with diabetes found that they are more susceptible to tau protein hyperphosphorylation [14]. These in vivo and in vitro outcomes point to a possible bidirectional link between T2D and AD risk.

Furthermore, impairment of brain insulin signalling and brain IR is associated with the development of mild cognitive impairment (MCI) [15]. However, insulin treatment did not reduce brain IR [15]. Conversely, insulin‐sensitising medications that overcome IR may be able to more successfully reduce brain IR and AD neuropathology. Belonging to the biguanide group, metformin is an insulin‐sensitising molecule that is used as a crucial therapeutic treatment for T2D by regulating hepatic gluconeogenesis and glycogenolysis [16]. The fundamental cause of peripheral and brain IR may be related to the dysregulation of the peroxisome proliferator‐activated receptor alpha (PPAR‐α) which regulates insulin sensitivity. Furthermore, PPAR‐α has been demonstrated to be significantly dysregulated in both T2D [17] and AD [18]. It has been established that augmentation of peripheral and central PPAR‐α by PPAR‐α agonist fenofibrate (FN) can attenuate the metabolic derangement in T2D and reduce the progression of AD [19, 20]. However, the underlying mechanism for the neuroprotective effect of FN against AD is not fully elucidated. Therefore, this review has been amended to discuss the possible role of PPAR‐α agonist FN in AD and T2D.

## 2 The Link Between T2D and AD

T2D is a metabolic disease characterised by insulin insensitivity and dysfunctional β cells in the pancreas, which results in hyperglycaemia and IR [21]. Of note, 90% of diabetes is T2D which often affects persons over the age of 40 [22]. Obesity, hypertension and dyslipidaemia are among the most cardiometabolic diseases associated with the development of T2D [16]. T2D patients may be asymptomatic and may be present with long‐term complications such as nephropathy, retinopathy and other microvascular complications [23]. However, the classical clinical features of T2D are polyuria, polydipsia, polyphagia and progressive weight loss [24].

T2D develops due to the progressive pancreatic β‐cell dysfunction [25]. Notably, amyloid formation in the pancreatic β cells leads to progressive pancreatic β‐cell death, pancreatic β‐cell dysfunction and the development of overt T2D advance [26]. Islet amyloid polypeptide (IAPP) which is commonly referred to as an amylin, is the primary component of pancreatic β‐cell amyloids, and is mostly cleaved by pancreatic neprilysin (NEP) [26]. Importantly, progressive deposition of IAPP in the pancreatic β cells is associated with pancreatic β‐cell toxicity and dysfunctions [26]. Pancreatic β‐cell dysfunction is prevented by NEP which cleaves and eliminates extracellular pancreatic IAPP. Therefore, inhibition of the pancreatic IAPP by activating NEP seems to be protective against the development of T2D [27].

Furthermore, the aggregation of Aβ in the brain facilitates the development of AD, whereas the accumulation of IAPP in the pancreas induces the development of T2D [28]. Pre‐pro‐IAPP is the precursor to IAPP, which is a 37‐amino acid peptide [28]. Notably, pancreatic β cells cosecrete insulin into the bloodstream together with IAPP. As IAPP builds up over time, oligomers and fibrils develop, and amyloid deposits are subsequently observed in T2D [28].

Moreover, IAPP‐induced pancreatic β cytotoxicity may be exacerbated by human IAPP processing inefficiency brought on by abnormalities in autophagy and proteasome activity [29]. Consequently, restoring IAPP proteostasis could be an effective approach to treat and prevent T2D [30]. Consequently, the pathophysiology of brain IR and cognitive dysfunction have similar characteristics in both T2D and AD. As a result, AD is categorised as type 3 diabetes (T3D) [31]. T3D refers to the metabolic syndrome that may lead to abnormalities linked to progressive brain IR with consequent impairment of central insulin signalling processes, accumulation of neurotoxins, neuronal stress and resulting in a course of neurodegeneration [31]. Many studies have specified that impaired hippocampus insulin signalling distorts memory and other executive functions, attributing to the decline of insulin signalling and concomitant development of IR [32, 33].

Furthermore, brain and peripheral IR lead to hyperglycaemia, which participates in the development of T2D‐linked comorbidities, such as obesity and dyslipidaemia [31]. Subjects with hyperglycaemia in AD present with neuronal loss, formation of plaques and tangles and reduced neurogenesis. Inflammation seems to play an essential role in the development of IR in AD and T2D. Alterations in glucose metabolism result from changes in the expression of the insulin receptor substrates 1 and 2 (IRS‐1 and IRS‐2), and seem to be mediated by several inflammatory pathways being present in both pathologies [32, 33].

Although there are some similarities in the IR of AD and T2D, brain and peripheral IR also have their distinct features. Failure to activate IRS‐1 is the hallmark of AD, while inhibition of IRS‐2 is the main feature of T2D. Inflammation mediates the alterations in glucose metabolism in AD and T2D. Targeting inflammation and insulin receptors may be a successful strategy to prevent and ameliorate T2D and AD symptoms [26, 27].

These verdicts highlighted that T2D and AD share several metabolic defects, such as IR, impaired glucose metabolism and mitochondrial defects. Prominently, T2D considerably increases the risk of cognitive decline and dementia, particularly AD. Besides, patients with AD are more susceptible to T2D and the possibility of linkage between the processes responsible for the loss of brain cells and β cells in these diseases [26, 27, 28].

## 3 Pharmacology of FN

FN is a chlorobenzophenone derivative drug used in the management of hypertriglyceridaemia, mixed dyslipidaemia and diabetic retinopathy and can reduce primary and secondary cardiovascular events [34, 35]. In 1974, FN was mostly synthesised from clofibrate in France under the name precetofen, which the WHO no‐proprietary guideline, renamed it as a FN [36]. FN is a lipid‐modifying drug mainly used in patients with elevated triglyceride levels. FN exerts its action via the activation of PPAR‐α. FN is a useful option for patients with primary combined dyslipidaemias or secondary dyslipidaemias, such as those associated with T2D and metabolic syndrome [36]. By activating PPAR‐α, FN modulates the expression and the functional activity of lipoprotein lipase and apolipoprotein CIII resulting in lipolysis and the removal of triglycerides from plasma [37, 38].

Apolipoproteins AI and AII are expressed more when FN is present, which lowers LDL and VLDL levels while raising HDL [37, 38]. Furthermore, regardless of glycaemic management, FN reduces the incidence of amputation by 37% in T2D patients [34, 35]. FN is susceptible to pharmacological interactions with immunosuppressive medications, statins, warfarin and bile acid sequestrants [37, 38]. The most frequent side effects of FN are headache, arthralgia, myalgia and kidney stones. Patients with hypothyroidism, hypersensitivity, liver illness, gallbladder disorders and renal impairment should not use FN [38, 39].

## 4 Role of FN in T2D and AD

### 4.1 FN in T2D

FN reduces cardiovascular events in T2D patients proposing the possible role of FN in preventing cardiovascular complications by reducing atherogenic dyslipidaemia [19]. FN is recommended in T2D patients with dyslipidaemia which develops due to dysregulation of peripheral insulin signalling [39]. FN also improves lipid profile in T2D patients with poor glycaemic control by shifting low‐density lipoprotein (LDL) towards nonatherogenic LDL [40]. A systematic review revealed that early use of FN in T2D patients prevents endothelial dysfunction and can attenuate other microvascular dysfunctions [41]. FN also has anti‐inflammatory effects and reduces cardiometabolic disorders in patients with metabolic syndrome [42]. A cohort study involving 242 patients with metabolic syndrome with or without T2D randomised to FN, atorvastatin and placebo illustrated that FN group experienced more glucose homeostasis than others [43]. This finding suggests that FN effectively attenuates metabolic syndrome mediated by prediabetes. Likewise, FN improves impaired glucose tolerance, including impaired fasting glucose and impaired glucose tolerance in patients with metabolic disorders [43]. Therefore, FN could be an adjuvant therapeutic modality in controlling glucose homeostasis in T2D patients. However, intensive blood pressure control and prolonged use of FN in T2D patients may increase the risk of kidney dysfunction, as evident by increased serum creatinine [44]. Different studies implicated FN in the deterioration of kidney function by increasing serum creatinine levels [45, 46]. Evidence from clinical studies illustrated that FN use within weeks increases serum creatinine levels [47]. It has been reported that prolonged use of 200 mg/day of FN increases creatinine production and serum creatinine level without any effect on the eGFR [44]. Nevertheless, this effect is only observed in patients with underlying kidney diseases or when used with drugs that alter kidney haemodynamics, such as renin–angiotensin (RAS) inhibitors [47]. The mechanism underlying the harmful effects of FN on renal function may involve the inhibition of vasodilator prostaglandin, and glomerular pressure and reduce creatinine clearance [47]. However, FN is effective in managing hypertensive glomerulosclerosis and diabetic nephropathy [47].

Regarding the potential role of FN in the regulation of glucose homeostasis, Holm et al. [42], confirmed that FN improves glucose homeostasis in mice by increasing sulfatide and long‐chain sphingolipids, which improve pancreatic islet insulin sensitivity. Moreover, FN improves blood glucose homeostasis by attenuating inflammation and apoptosis in the pancreatic β cells [36]. An experimental study demonstrated that administration of FN in mice increases the size of sympathetic neurons and tyrosine hydroxylase activity in the pancreatic islet cells through enhancement of pancreatic β function [42]. Remarkably, FN can reverse autoimmune response in nonobese diabetic mice by augmenting the level of sulfatide and long‐chain sphingolipids in the pancreatic islet with subsequent improvement of pancreatic insulin sensitivity [48]. Different experimental studies revealed that sulfatide and long‐chain sphingolipids improve pancreatic β cells [49, 50]. Sulfatide and long‐chain sphingolipids are regarded as immune modulators and regulate insulin secretion from pancreatic β cells [51]. Furthermore, FN protects against liver lipogenesis and hepatic IR by inhibiting the development of endoplasmic reticulum (ER) stress [52].

Moreover, PPAR‐α is essential in the expression of hepatokines which involve the regulation of nutrition in mice [53]. Notably, hepatokines which are hepatic‐derived proteins improve glucose homeostasis [54]. Sparc‐related modular calcium‐binding protein 1 (SMOC1) regulates glucose homeostasis in mice and might be a therapeutic target in managing T2D [54]. Circulating SMOC1 is positively correlated with insulin sensitivity [55]. Hepatokine fibroblast growth factor 21 (FGF21) is improved by long‐term FN therapy, and this raises insulin sensitivity in T2D [56]. As a result, FGF21 appears to be a possible target for T2D therapy [57]. Furthermore, T2D and the development of the metabolic syndrome are predicted by FGF21 resistance [58]. It has been documented that patients with impaired glucose tolerance and T2D have increased levels of circulating FGF21 as a compensatory mechanism [59].

**FIGURE 3:**
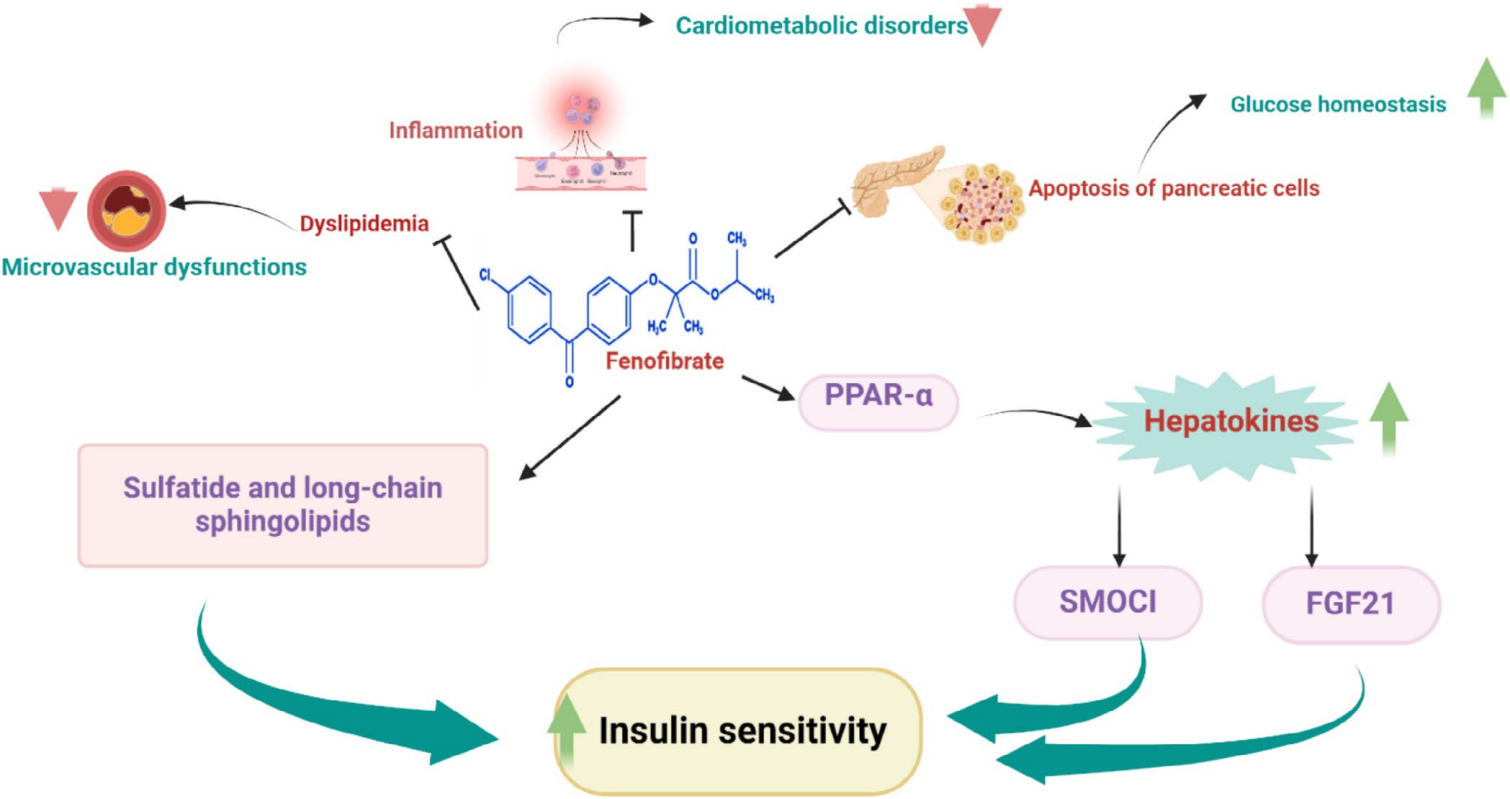
The role of FN in T2D: FN by activating PPAR‐α promotes the release of hepatokines which increase the expression of fibroblast growth factor 21 (FGF21) and Sparc‐related modular calcium‐binding protein 1 (SMOC1) that increase insulin sensitivity. In addition, FN by increasing the release of sulfatide and long‐chain sphingolipids augments insulin sensitivity.

These findings suggest that FN mitigates IR, improves pancreatic β cells and could be a therapeutic strategy in the management of T2D (Figure 3).

### 4.2 FN in AD

The distribution of brain PPAR‐α was initially recognised by Warden and Truitt [20] by immunofluorescence. PPAR‐α is expressed in all cell types in mice and humans [20]. PPAR‐α promotes the expression of N‐methyl‐D‐aspartate (NMDA) receptors, and adenosine monophosphate protein activated (AMPA) receptor‐associated glutamate receptor, which improves synaptic plasticity via cyclic response element binding protein (CREBP) [60]. PPAR‐α regulates glutamate neuronal homeostasis by increasing the expression of glutamate transporters in astrocytes [61]. Glutamate transporters in astrocytes are downregulated by the presence of Aβ in AD, leading to postsynaptic overstimulation and neurotoxicity [61]. Originally, it has been established that FN through activation of PPAR‐α inhibits beta‐site amyloid precursor protein enzyme 1 (BACE‐1), thereby reducing soluble APPβ and Aβ_42_ release [62]. However, FN has no inhibitory effects on the presenilin‐1 (PS‐1) and APP [62]. Thus, FN affects the amyloidogenic process and may reduce AD neuropathology. FN is regarded as an inverse γ‐secretase modulator which increases the generation of Aβ_1‐42_ [63]. Also, FN enhances the clearance of Aβ_1‐42_ [63]. Nonetheless, FN, through modulation of γ‐secretase, promotes the production of highly aggregated Aβ_42_ and reduces Aβ_38_ production [64]. In this sense, FN and selective cyclooxygenase (COX‐2) inhibitors, coxibs, were considered as diverse compounds that induce AD development in animal model studies [64]. Even though Aβ is the primary pathology of AD in preclinical studies, most clinical trials failed to confirm this hypothesis [18]. Therefore, the intervention of early risk factors involved in AD neuropathology such as apolipoprotein E4 (ApoE) by PPAR‐α activators may reduce AD risk [18].

Notably, PPAR‐α is intricate in various brain disorders, including depression, schizophrenia, sleep disorders, stroke and epilepsy, through modulation of hippocampal brain‐derived neurotrophic factor (BDNF), endocannabinoid and cholinergic signalling [18]. In addition, PPAR‐α agonists such as Wy14643 improve cognitive function and prevent scopolamine‐induced cognitive dysfunction in mice [65]. A previous study by de la Monte observed that PPAR‐α was downregulated in AD brains [66], suggesting an involvement of PPAR‐α in AD neuropathology. Notably, endogenous Aβ production is augmented in PPAR‐α knockout mice [67], suggesting a protective role of PPAR agonists against AD neuropathology. Besides, PPAR‐α agonists have been reported to produce beneficial effects in many preclinical AD models [68, 69]. PPAR‐α agonist gemfibrozil attenuates Aβ‐induced neurotoxicity and burden with a reduction of astrogliosis and microgliosis in the hippocampus and cortex of mice [68]. Likewise, cinnamic acid, a potent PPAR‐α activator, inhibits cognitive impairment in the AD mouse model [68]. Cinnamic acid upregulates transcription factor EB (TFEB) and stimulates lysosomal biogenesis. Moreover, cinnamic acid reduced cerebral amyloid plaque burden and improved memory via PPARα [68]. Therefore, stimulation of lysosomal biogenesis by cinnamic acid may have therapeutic implications for the treatment of AD and other lysosomal disorders originating from accumulation of toxic protein aggregates. In particular, PPAR‐α agonists, Wy14643, and gemfibrozil enhance the cognitive abilities of AD mice models by augmentation of autophagosome biogenesis [70]. Gemfibrozil and Wy14643 induce autophagy in human microglia cells and U251 human glioma cells stably expressing the human APP and this effect is PPARα‐dependent. Administration of PPARα agonists decreases amyloid pathology and reverses memory deficits and anxiety symptoms in APP‐transgenic mice. There is a reduced level of soluble Aβ and insoluble Aβ in hippocampus and cortex tissues from APP transgenic mice after treatment with either gemfibrozil or Wy14643, which promoted the recruitment of microglia and astrocytes to the vicinity of Aβ plaques and enhanced autophagy [70]. These results indicated that PPARα is an important factor regulating autophagy in the clearance of Aβ and suggested gemfibrozil be assessed as a possible treatment for AD. A clinical trial conducted in 2020 that evaluated the possible protective effect of gemfibrozil on AD neuropathology in subjects with normal or mild cognitive impairments illustrated that gemfibrozil improves cognitive function [18]. Gemfibrozil has been shown to increase mir‐107, which is involved in the regulation of BACE‐1 [71]. In AD patients, mir‐107 is reduced, causing activation of BACE‐1 and progressive Aβ production and advancement of AD neuropathology [71]. Interestingly, a disintegrin and metalloproteinase domain‐containing protein 10 (ADAM10), commonly known as α‐secretase, is activated by gemfibrozil and other PPAR‐α agonists, triggering the neuroprotective nonamyloidogenic pathway [67]. Hippocampal integrity and synaptic plasticity are enhanced by the neuroprotective soluble APPα that is produced when α‐secretase cleaves APP [72]. Thus, PPAR‐α agonists can decrease AD neuropathology by shifting APP proteolysis towards the nonamyloidogenic pathway. In addition, activation of PPAR‐α by other agents such as low dose of aspirin and statins induces a significant reduction of Aβ‐induced neurotoxicity and AD neuropathology [73, 74]. Of note, aspirin is one of the most widely used medications in the world, upregulates TFEB and increases lysosomal biogenesis in brain cells. Excitingly, aspirin induced the activation of PPAR‐α and stimulated the transcription of *TFEB* gene via PPARα. Oral administration of low‐dose aspirin decreased amyloid plaque pathology in both male and female transgenic mice in a PPARα‐dependent fashion [70]. The inhibitors of HMG‐CoA reductase and cholesterol‐lowering drugs, statins, were found to excite the expression of neurotrophins in brain cells independent of the mevalonate pathway. Statins serve as ligands of PPARα and that Leu331 and Tyr 334 residues of PPARα are important for statin binding. Statins increase neurotrophins through PPARα‐mediated transcriptional activation of CREB signalling. Accordingly, simvastatin increases CREB and brain‐derived neurotrophic factor (BDNF) in the hippocampus of mice [71]. Furthermore, metformin inhibits DPP4 circulating level and increases glucagon‐like peptide 1 (GLP‐1) via PPARα and is independent of AMPK in mice. Thus, metformin exerts glucoregulatory actions through modulation of the incretin axis [75]. It has been shown that myocardial lipid accumulation, oxidative stress, apoptosis and cardiac remodelling and dysfunction induced in T2D by low streptozotocin doses and high‐fat diets were considerably reversed by GLP‐1 agonists and analogues treatments for 8 weeks in mice [76]. GLP‐1 protects cardiac function by inhibiting the ROCK/PPARα pathway, thereby ameliorating lipotoxicity in diabetic cardiomyopathy [76]. Both metformin and GLP‐1 agonists and analogues have neuroprotective effects against the development and progression of AD [77, 78].

Remarkably, PPAR‐α agonists improve synaptic plasticity in male mice only due to higher expression of brain PPAR‐α in males [79]. PPARα mediates the improvement of hippocampal synaptic plasticity upon nuclear retinoid X receptor (RXR) activation in a transgenic mouse model with cognitive deficits. This improvement results from an increase in GluA1 subunit expression of the AMPA receptor, eliciting an AMPA response at the excitatory synapses. Associated with a two times higher PPARα expression in males than in females, thus; male, but not female, PPARα null mutants display impaired hippocampal long‐term potentiation [79]. Moreover, PPARα knockdown in the hippocampus of cognition‐impaired mice compromises the beneficial effects of RXR activation on synaptic plasticity only in males. Furthermore, selective PPARα activation with pemafibrate improves synaptic plasticity in male cognition‐impaired mice, but not in females [79]. Interestingly, hormones are known to influence the expression of PPARα in a sex‐specific manner because gonadectomy of male rats decreases PPARα expression levels. Oestrogens are known to improve synaptic plasticity, and behaviour is affected in ovariectomised female rats [80]. Thus, PPAR‐α agonist FN enhances synaptic plasticity in male but not in female mice [79], suggesting a sexual dimorphism in response to PPAR‐α agonists in AD management, and this may explain a higher AD risk in women [81]. Of interest, both oestrogen and androgen improve PPAR‐α expression [82, 83]. Oestrogen replacement therapy in menopausal women improves cognitive function and reduces AD risk by increasing the expression of neuronal PPAR‐α [84].

These mouse models do not fully reflect all pathological changes observed in patients and translating synaptic plasticity changes in mice with cognitive deficits in humans is challenging. However, a PPARα agonist bexarotene improves cognition in mouse models, and in a patient with mild AD [85].

Moreover, PPAR‐α agonist FN regulates the endogenous inhibitor of glutamate kynurenic acid, which is derived from L‐tryptophan and involved in memory impairment [86, 87]. PPAR‐α agonist gemfibrozil improves glutaminergic neurotransmission and associated memory function by inhibiting the synthesis of kynurenic acid [88, 89]. Also, PPAR‐α increases the expression of nicotinic acetylcholine receptor alpha 7 (nAChR‐α7) [90], which is downregulated in AD [91]. Notably, nAChR‐α7 is highly expressed in the cerebral cortex and hippocampus, mainly in the presynaptic and postsynaptic neurons [91]. In AD, nAChR‐α7 is highly dysregulated by Aβ, which is disseminated on the cell membrane via the internalisation of these receptors [91]. The interaction between nAChR‐α7 and Aβ leads to inhibition and stimulation at different brain regions causing abnormal neurotransmission in AD model [92]. However, the main effect of Aβ is inhibitory on the nAChR‐α7 since nAChR‐α7 agonists improve cognitive function in animals treated by Aβ [93].

In addition, PPAR‐α signalling phosphatidylinositol 3 kinase (PI3K) inhibits the Aβ production [94]. PI3K pathway has a neuroprotective effect against AD neuropathology. Notably, nAChR‐α7 through activation of the PI3K pathway inhibits Aβ‐induced neurotoxicity [95]. Interestingly, the universal PPAR activator GFT1803 inhibits production and increases the clearance of Aβ, though PPAR‐α agonists are more effective in reducing Aβ production [96].

Moreover, PPAR‐α regulates various cellular processes, including oxidative stress, mitochondrial dysfunction and neurotransmission dysfunction, mainly in the amygdala and prefrontal cortex [97]. Therefore, PPAR‐α agonists may be recommended in the treatment of neurodegenerative disorders, including AD. PPAR‐α agonist FN has been reported to be effective in different neurodegenerative disorders including PD, MS and amyotrophic lateral sclerosis (ALS) [97]. For example, PPAR‐α agonist FN reduces cognitive dysfunction in preclinical and PD patients [98]. Of interest, FN reduces depressive symptoms through the modulation of neurosteroids [99] that are common in different neurodegenerative disorders, including AD.

**FIGURE 4:**
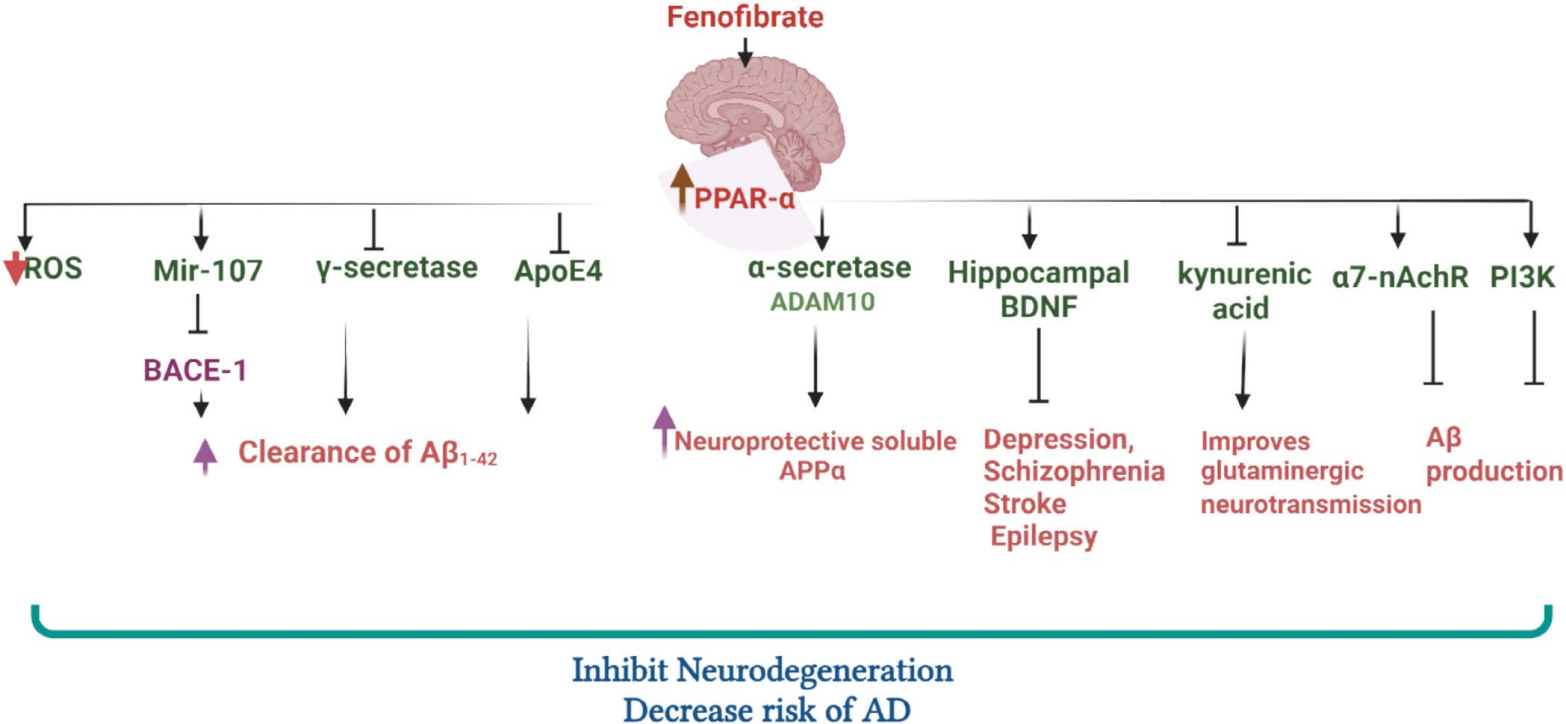
Role of FN in AD: FN attenuates AD neuropathology by activating PPAR‐α which through modulation of many signalling pathways and neurotrophic factors can inhibit the production and augment the clearance of Aβ. Also, FN activates the neuroprotective nonamyloidogenic pathway by activating α‐secretase. For detail see the text above.

Taken together, PPAR‐α agonist FN seems to be effective in both AD and T2D. However, the underlying mechanistic role of FN in these conditions needs to be elucidated according to cellular and molecular pathways (Figure 4).

## 5 Mechanistic Role of FN in AD and T2D

### 5.1 Immunoinflammatory Response

It has been shown that abnormal immunoinflammatory response could be the possible mechanism linking T2D and AD [100]. IR and the pathophysiology of T2D are considerably influenced by systemic inflammation. In general, microglia respond to peripheral inflammation in an adaptable manner. After exposing mice to IL‐1β and TNFα systemically, the hippocampus exhibits the production of cytokines and chemokines [25, 101, 102, 103]. It has been illustrated that immune system stimulation in mice causes brain pathologies similar to AD, which include the accumulation of APP and its proteolytic fragments as well as changes in tau phosphorylation [104]. Higher peripheral amounts of IL‐6, TNF‐α, IL‐1β, TGF‐β, IL‐12 and IL‐8 are linked with AD [105]. Patients with AD who are in the early stages of the disease have activated peripheral immune cells in circulation. Peripheral inflammation and cognitive dysfunction have also been related in a number of studies. Peripheral inflammatory indicators, for instance, have been seen in AD patients [106]. Increased peripheral TNF‐α and IL‐1β levels have been linked to an increased risk of AD [106]. It has been demonstrated that proinflammatory cytokines can cross the BBB [107]. Gene expression analysis has demonstrated that brain‐specific inflammatory responses are triggered by peripheral inflammation [107]. An increase in central inflammation is also likely to result from harm to the BBB and subsequent immune cell infiltration. It is unclear whether cerebral inflammation develops from peripheral inflammation or the other way around [106, 107]. These findings highlighted that peripheral and central immunoinflammatory responses are intricate in the development and progression of both AD and T2D.

By modifying the immunological response of T cells, FN can reduce the autoimmune response in mice with experimental Sjogren syndrome [108]. Mice lacking in PPAR‐α exhibited aberrant immunological reactions to the inflammatory mediators such as leukotrienes and prostaglandins [22, 109, 110, 111, 112, 113]. PPAR‐α ligands reduce the expression of adhesion molecules, cyclooxygenase‐2 (COX‐2) and IL‐6 [114]. By upregulating the production of NF‐κB inhibitor (IκBα), PPAR‐α ligands suppress the expression of NF‐κB [115]. It has been shown that the PPAR‐α ligand WY14643 prevents IgG from interacting with myelin oligodendrocytes in mice. FN and other PPAR‐α ligands reduce inflammation by promoting the release of anti‐inflammatory cytokines [115, 116]. Remarkably, PPAR‐α ligands increase the production of Th2 cytokines [117]. FN is able to decrease Th17 differentiation compared to other PPAR‐α agonists such as bezafibrate. FN inhibits the expression of STAT3 and IL‐21, both of which are necessary for Th17 differentiation [23, 98, 118, 119, 120, 121]. A case–control study revealed that prolonged use of FN lowers inflammatory markers, such as CRP and IL‐6 in patients with dyslipidaemia [122].

Notably, COX‐2 upregulation is associated with vascular inflammation and impairment of vasomotor response in T2D [123]. Targeting of COX‐2 by specific inhibitors attenuates the development of long‐term complications such as diabetic neuropathy [124]. Similarly, IL‐6 serum level is augmented in obese T2D patients compared to healthy controls [125]. In addition, NF‐κB is upregulated and associated with T2D complications in both animals and humans [126]. Therefore, Th2 cytokine production is impaired in T2D patients leading to abnormal immune response and immunoinflammatory response [127]. Overactivated NF‐κB is engaged with the development of synaptic dysfunction in diabetic encephalopathy [128]. Consequently, an excess of inflammation coupled with an aberrant immune response may represent a potential connection between AD and T2D [129]. In addition, COX‐2 expression is elevated in AD and linked with the development of neuroinflammation, thus; inhibiting COX‐2 may be helpful in the treatment of AD [130]. Compared to healthy controls, AD patients had higher serum levels of IL‐6 [131]. Dysregulation of the anti‐inflammatory Th2 immune response may be the cause of this aberrant immunoinflammatory response in AD patients. Furthermore, the pathophysiology of both AD and T2D involves the nod‐like receptor pyrin 3 receptor (NLRP3) inflammasome [132] which is involved in the pathophysiology of both AD and T2D [133, 134]. The maturation of IL‐1β and IL‐18 as well as the activation of caspase‐1 are mediated by the NLRP3 inflammasome [133, 134]. NF‐κB is one of the several triggers that activate the expression of the NLRP3 inflammasome [135, 136, 137, 138, 139]. The pathophysiology of neuroinflammation and neurodegeneration are intricately linked to the activation of NLRP3 inflammasome [133]. Additionally, NLRP3 inflammasome is amplified and associated with the severity of AD [134]. Within activated microglia, the NLRP3 inflammasome stimulates the production and release of IL‐1β and IL‐18. The pathophysiology of AD is linked to abnormal activation of the NLRP3 inflammasome, as demonstrated by preclinical and clinical results [134]. Elevations of IL‐1β CSF levels in patients with severe AD indicate an overactivation of the NLRP3 inflammasome [134]. AD severity can be attenuated by using certain inhibitors to target the NLRP3 inflammasome [134]. Importantly, FN inhibits the production of NLRP3 inflammasome and NF‐κB, thus; it has a strong anti‐inflammatory effect against the development of lung inflammation [140]. By blocking the NF‐κB signalling pathway, FN prevents retinal damage and injury of the retinal blood barrier [141]. Additionally, by suppressing the expression of NLRP3 inflammasome, FN can decrease the severity of diabetic retinopathy [141].

Furthermore, innate immune sensors known as toll‐like receptors (TLRs) alert the immune system to the presence of exogenous pathogens [100]. When TLR is activated, the proinflammatory cytokines are released, and the adaptive immune system is activated to get rid of invasive pathogens [100]. TLR is capable of identifying danger signals that result from tissue damage and inflammation. TLRs play a critical role in AD neuropathology and are abundantly expressed by immune cells in the CNS [142]. TLR agonists contribute to the exaggeration of detrimental inflammatory reactions. Since PPAR‐α activation inhibits TLR expression through many pathways, it has been proven that PPAR‐α agonists and TLRs have reciprocal interactions [143]. FN suppresses CD14 expression which raises TLR expression and causes the release of pro‐inflammatory cytokines [144]. Furthermore, FN suppresses the expression of the MyD88‐TLR4 signalling pathway and the release of IL‐12 [145]. TLRs are greatly upregulated in T2D, contributing to the advancement of inflammatory diseases and associated diabetic complications [146]. TLRs may, therefore, be a possible connection between AD and T2D. Thus, by blocking TLRs and their effectors, the PPAR‐α agonist FN can suppress the first immunological response in AD neuropathology.

Therefore, FN through modulation of the immunoinflammatory signalling pathway can mitigate the pathogenesis of T2D and AD neuropathology.

### 5.2 Neuroinflammation

The development of various neurodegenerative diseases is linked with the development of neuroinflammation [147]. Neuroinflammation is created by the activation of T and B lymphocytes in the CNS [148]. For instance, synaptopathy caused by neuroinflammation during the early stages of MS can occur independently of the demyelination process, which could account for cognitive failure in MS patients [149]. Exaggerated immunological disruption and the progression of neuroinflammation in the late stages of MS contribute to the pathophysiology of the disease [149, 150]. Patients with AD have reduced cholinergic activity, which controls immune cell response and activity [151]. When immune cells' levels of acetylcholine are lowered, the proinflammatory cytokines are released leading to the development of neuroinflammation [151, 152]. Thus, reducing neuroinflammation may be a useful therapeutic approach to reduce the neuropathology associated with AD. A low‐grade chronic inflammatory disease accelerates the development of neuroinflammation in T2D [153]. Inhibition of the proinflammatory cytokines and the inflammatory signalling pathway has been shown in an experimental study to reduce the development of neuroinflammation in diabetic mice [154]. Hyperglycaemia‐induced BBB injury promotes the transport of inflammatory cells and the development of neuroinflammation in T2D [155].

**FIGURE 5:**
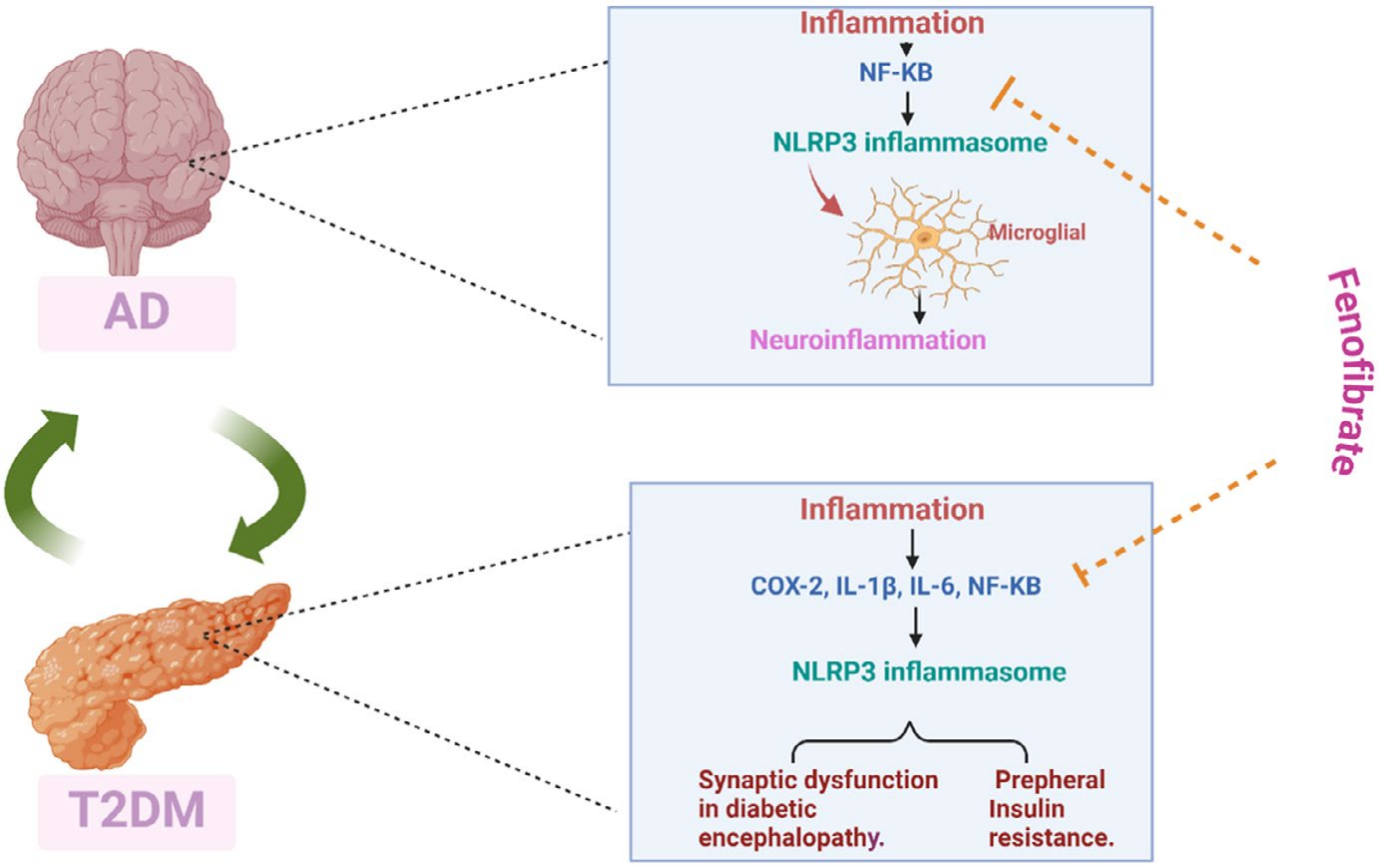
Role of inflammation in AD and T2D: FN inhibits the expression of inflammatory signalling pathways such as NF‐κB and NLRP3 inflammasome, suppresses the release of proinflammatory cytokines and inflammatory molecules which implicated in the development of neuroinflammation in both AD and T2D.

Various preclinical studies have demonstrated the protective effect of FN against the onset and progression of neuroinflammation [156, 157]. In rats with severe brain damage, FN reduces neuroinflammation by inhibiting the development of oxidative stress [134]. By preventing oxidative stress, neuroinflammation and mitochondrial dysfunction, the risk of neurodegeneration is reduced. Importantly, FN exerts a neuroprotective impact against the onset and progression of MS by suppressing oxidative stress, neuroinflammation and mitochondrial dysfunction [157]. The suppression of inflammatory signalling pathways as well as antioxidant and anti‐inflammatory actions are the fundamental mechanisms by which FN contributes to the inhibition of neuroinflammation [156, 157]. FN inhibits neuroinflammation by activating neurons nicotinic cholinergic receptors [158]. These results suggested that FN may modify neuroinflammatory responses to lower the aetiology of AD and T2D (Figure 5).

### 5.3 Oxidative Stress

Oxidative stress plays a crucial role in the development of AD by increasing neuronal injury and apoptosis [16, 138, 159, 160]. It has been revealed that reactive oxygen species (ROS) which facilitate the activation of microglia can induce neuronal injury and apoptosis [161]. In AD, inflammatory responses can trigger the development of oxidative stress in the activated macrophages and microglia, resulting in neuronal injury [162]. Of interest, oxidative stress and the generation of ROS contribute to the progression of inflammation in AD [161]. As a result, oxidative stress and inflammation are positively activated in a vicious cycle in AD. According to these results, oxidative stress may intensify inflammatory responses, increase neuronal damage and accelerate the progression of AD neuropathology [161]. Consequently, using of antioxidants may prevent AD development and progression. Interestingly, antioxidants may be advantageous for human AD by reducing oxidative stress and associated inflammatory changes [163]. For example, the antioxidant alpha lipoic acid has been shown in preclinical and clinical trials to attenuate brain atrophy and enhance the clinical course of AD [164]. Similarly, oxidative stress is increased in T2D due to IR, redox imbalance and hyperglycaemia [165]. Furthermore, ER stress induced by hyperglycaemia boosts the production of ROS, which damages pancreatic β cell function and causes hyperglycaemia in a positive feedback loop [165]. Moreover, ROS production is increased by IR‐induced lipotoxicity, which leads to pancreatic β‐cell dysfunction [165]. NADPH oxidase activity and ROS were shown to be greater in the monocytes of T2D patients compared to controls [166]. Thus, oxidative stress is implicated in the aetiology and consequences of T2D.

Furthermore, PPAR‐α agonists have been demonstrated to have strong antioxidant properties and may be able to treat a variety of neurodegenerative diseases including AD [167]. In AD mouse models, lipid peroxidation, oxidative stress and inflammation are inhibited by the PPAR‐α agonist GW7647 [167]. In addition, FN increases antioxidant capacity and reduces oxidative damage brought on by hyperglycaemia [168]. Similarly, a PPAR‐α agonist clofibrate, reduces neuroinflammation and oxidative stress in AD rat model [169]. Furthermore, lipid peroxidation, oxidative stress and inflammation are inhibited by the PPAR‐α agonist GW7647 in T2D model [170].

**FIGURE 6:**
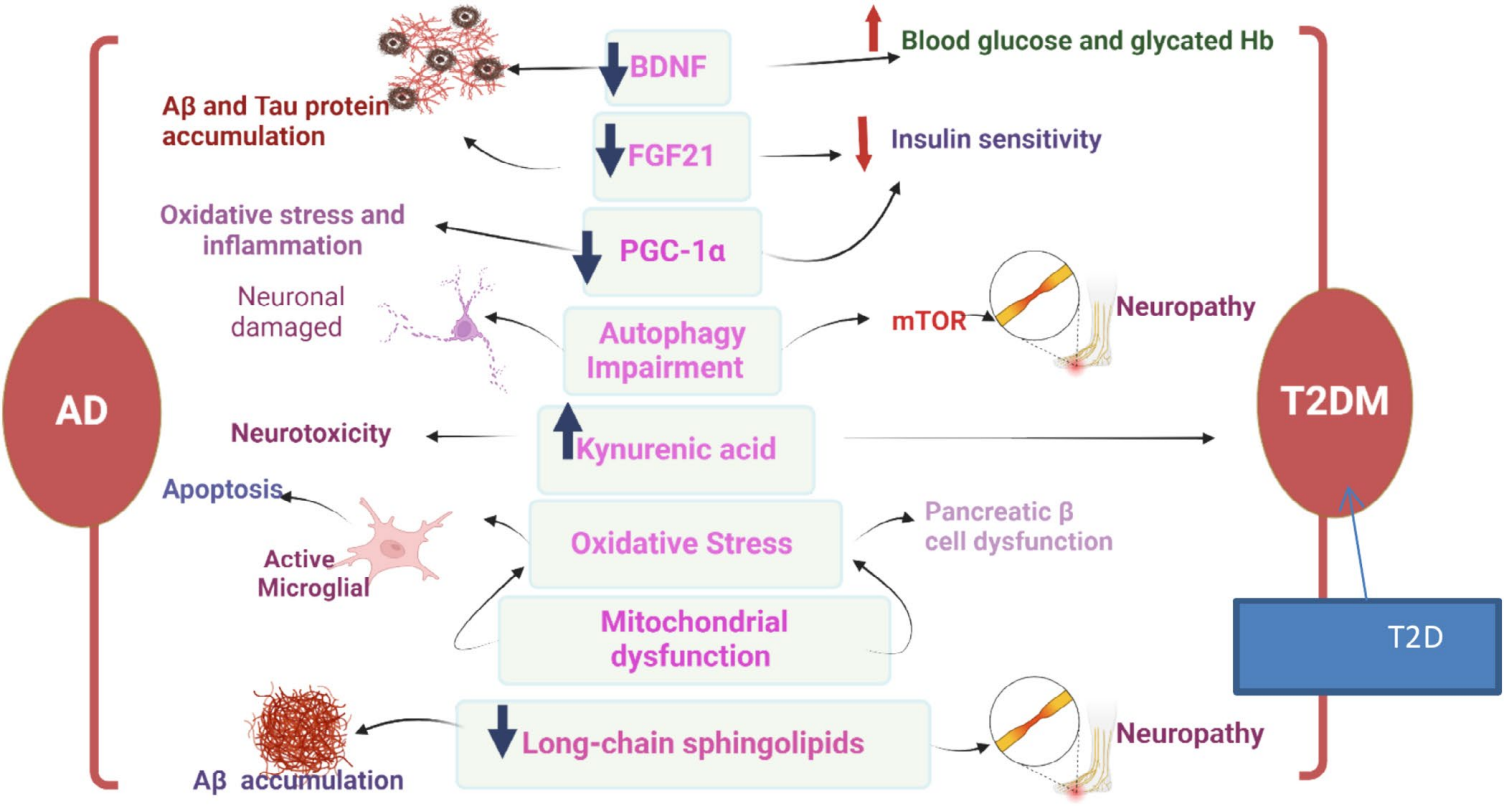
The crosstalk between AD and T2D: Many signalling pathways, cellular pathways and neurotrophic factors are dysregulated in both T2D and AD. For detail see the text.

Thus, FN can reduce the pathophysiology of AD and T2D by suppressing oxidative stress and enhancing endogenous antioxidant capacity (Figure 6).

### 5.4 Mitochondrial Dysfunction

Mitochondrial dysfunction plays a crucial role in the degeneration of neurons and axons in AD [171, 172]. The primary mechanism behind the development of mitochondrial dysfunction in AD is the impairment of mitochondrial permeability transition pores by Ca^2+^ dyshomeostasis and ROS [171, 173]. In AD, mitochondrial failure is thought to be a significant initiator of programmed neuronal death [174]. Serum lactate and uric acid are thought to be possible indicators of mitochondrial malfunction [175]. The aetiology of T2D also includes mitochondrial dysfunction [176]. Mitochondrial dysfunction is caused by changes in mitochondrial DNA due to ageing, obesity and dyslipidaemia [176]. Mitochondrial dysfunction is correlated with the emergence of IR, ER stress and ROS release [177]. Consistently, mitochondrial oxidative stress in the peripheral lymphocytes is higher in T2D patients compared to healthy controls [178].

Therefore, mitigating mitochondrial dysfunction could be a mechanistic approach to halt the progression of both T2D and AD. PPAR‐α agonists play a fundamental role in regulating mitochondrial function in diabetic patients [179]. Interestingly, FN improves insulin sensitivity by enhancing mitochondrial β‐oxidation [180]. Additionally, FN inhibits mitochondrial dysfunction in burn patients [179]. Moreover, FN promotes neurogenesis by modulating mitochondrial biogenesis in the experimental ischemic reperfusion injury [181]. The protective effect of FN against mitochondrial dysfunction is achieved by increasing the expression of mitochondrial uncoupling protein 2, which shields mitochondria from oxidative stress by reducing the production of ROS [181]. Consequently, FN shows potential efficacy in combating AD and T2D by suppressing mitochondrial dysfunction.

### 5.5 Autophagy

Autophagy is an essential process within cells that eliminate harmful misfolded proteins and damaged organelles. Impaired neuronal autophagy triggers the development and progression of neurodegeneration [182, 183, 184, 185]. In addition, autophagy regulates both adaptive and innate immunity, and any anomaly in autophagy can lead to an aberrant immunological response [186]. The survival and homeostasis of neurons mostly rely on basal neuronal autophagy. Malfunctioning autophagy plays a role in the development of AD [187]. Administering an early blockade of the mechanistic target of rapamycin (mTOR) a negative regulator of autophagy effectively decreases AD neuropathology in mice. Nevertheless, the suppression of mTOR during the later stage of AD worsens the neuropathology of the disease [188]. The process of autophagy can have both beneficial and harmful effects [188]. For instance, enhancing autophagy in T and B cells stimulates the progression of neuroinflammation, while suppressing autophagy in this context could potentially be a beneficial approach in the treatment of AD [188]. Surprisingly, restoration of basal neuronal autophagy function in late AD impedes the progression of disease severity [188, 189]. The autophagy process is also dysregulated in T2D as it increased in T2D compared to healthy controls [190]. The autophagy process in T2D is upregulated as a compensatory mechanism in response to IR, hyperglycaemia and proinflammatory cytokines [191]. Therefore, inhibition of the autophagy process and its regulator mTOR may attenuate T2D‐mediated complications [192]. However, an updated study illustrated that autophagy comprises T2D and ageing. Loss of autophagy promotes the development and progression of diabetic complications. Thus, activation of autophagy by miRNA may be a novel therapeutic option in the management of T2D and related complications [193].

These findings suggest a controversy regarding the potential role of autophagy in AD and T2D. Targeting of neuronal autophagy is crucial for understanding the neuropathology of AD. FN enhances the autophagy process, according to multiple studies [194, 195]. For instance, FN increases SIRT1 expression and autophagy function to mitigate myocardial damage in diabetic rats [194]. Additionally, FN inhibits acute kidney injury by controlling the autophagy process by inducing the expression of AMPK [99]. Both SIRT1 and AMPK activate autophagy process [196, 197]. Therefore, FN may slow the development of AD and T2D by activating autophagy process.

### 5.6 Brain‐Derived Neurotrophic Factor (BDNF)

BDNF is a growth factor produced and released from glial cells and neurons. BDNF controls synaptic plasticity, neurotransmission and neuronal development and maturation [198, 199, 200, 201, 202, 203]. BDNF acts by activating tropomyosin receptor kinase B (TrkB) receptor which is widely expressed in the CNS. BDNF has a neuroprotective effect against many neurodegenerative diseases including AD [204]. Neuroinflammation, tau protein phosphorylation, neuronal death and Aβ inhibit BDNF signalling. BDNF/TrkB induces PI3K and improves synaptic function by modifying intracellular glutamate and Ca^2+^ neurotransmission [204]. Notably, Aβ accumulation interferes with BDNF/TrkB signalling which is extremely dysregulated in AD [153, 204]. BDNF and pro‐BDNF are reduced in the human brains in early AD neuropathology [205]. Ng et al. [206] found that BDNF serum levels were reduced in AD patients compared to the healthy controls. Similarly, CSF BDNF levels were lower in AD patients compared to the healthy controls [207]. Furthermore, the severity of cognitive impairment is correlated with low serum levels of BDNF [208].

On the other hand, BDNF regulates insulin sensitivity and glucose homeostasis [209]. A case–control study showed that hyperglycaemia blocks BDNF output from the brain in T2D and healthy controls [209]. In addition, BDNF is expressed in many no‐neuronal tissues, including pancreatic α cells and cardiomyocytes [210]. Platelets are the main source of peripheral BDNF in T2D [210]. BDNF reduces blood glucose and glycated Hb by central inhibiting of food intake and through the peripheral effect by impeding glucagon release from the pancreatic α cells [211, 212]. Mutation of BDNF/TrkB signalling induces the development of obesity and IR [213]. Also, the reduction of circulating BDNF is correlated with a reduction in insulin sensitivity [214]. These results highlighted that BDNF is decreased in T2D and augmentation of BDNF may reduce IR and T2D‐related complications.

Furthermore, FN reduces the impact of hippocampal ischemia–reperfusion and enhances cognitive performance in rats [215]. The neuroprotective effect of FN is mediated by upregulating the expression of BDNF [216]. Additionally, PPAR‐α agonists increase the expression of hippocampus BDNF, which reduces the progression of neurodegeneration in neurodegenerative diseases [216]. BDNF controls the activity of microglia and prevents microglia‐induced neurodegeneration [217]. Specifically, naturally occurring phytochemicals such as curcumin, cannabinoids and genistein have been found to inhibit the progression of neurodegenerative disorders by upregulating the expression of BDNF [218]. Moreover, researches have been demonstrated that PPAR‐α agonists can enhance the treatment of T2D by increasing the activity of BDNF [219].

These data indicate that PPAR‐α agonist FN can decrease the development of AD neuropathology and T2D pathogenesis by enhancing the production of BDNF.

### 5.7 Fibroblast Growth Factor (FGF21)

FGF21 is a metabolic hormone also known as hepatokine that is produced and secreted by hepatocytes [220]. FGF21 regulates lipid and glucose metabolism by inducing fatty acid oxidation and gluconeogenesis, respectively, and it is abundantly expressed in the pancreatic islets and adipocytes [220, 221]. FGF21 regulates PPAR‐α expression in a feedback loop to improve insulin sensitivity [221, 222, 223, 224]. FGF21 has been shown by Chen et al. [225], to reduce the onset and progression of certain neurodegenerative diseases, such as AD. Results from studies conducted in vitro and in vivo demonstrated that FGF21 by upregulating protein phosphatase 2 A (PP2A) has a neuroprotective effect against oxidative stress and apoptosis caused by Aβ and tau protein [225]. Defective and aberrant autophagy triggers the release of FGF21 as a compensatory mechanism to modulate the autophagy process in AD [150]. Larson and Gill [226] demonstrated that FGF21 signalling was dysregulated in both AD and cardiometabolic disorders such as T2D, suggesting that FGF21 could be a possible link between AD and T2D. It has been shown that long‐term FN treatment improves FGF21, which enhances insulin sensitivity in T2D [56]. Therefore, FGF21 appears to be a potential therapeutic target for T2D [57]. Furthermore, the development of T2D and the metabolic syndrome are predicted by the development of FGF21 resistance [58]. There have been reports that T2D patients and impaired glucose tolerance had elevated levels of circulating FGF21 as a compensatory strategy [59]. Interestingly, a case–control study showed that FGF21 serum levels were shown to be lower in T2D patients compared to healthy controls [227]. Furthermore, the use of FN in those patients increased FGF21 levels in T2D patients. These findings pointed out that FGF21 is reduced in both AD and T2D patients. Therefore, increasing FGF21 by FN may improve these conditions or at least attenuate AD risk in T2D patients.

Moreover, AD and T2D are highly prevalent ageing‐related diseases associated with significant morbidity and mortality [228, 229, 230]. Some findings in human and animal models have linked T2D to AD‐type dementia. Despite epidemiological associations between T2D and cognitive impairment, the inter‐relational mechanisms could be related to the metabolic derangement. T2D is associated with cardiovascular and metabolic disorders which augment AD neuropathology. Therefore, there is an overlap between the pathologic mechanisms of T2D and AD [228, 229, 230]. The nexus of cardiometabolic risk factors, collectively referred to as the metabolic syndrome, has insulin resistance and hypertriglyceridaemia at its centre, and has been associated with the development of major types of dementia, including AD [231]. Triglyceride‐rich lipoprotein is involved in the development of AD in T2D patients by inducing the expression of brain ApoE4 [231]. Notably, FN is effective in treating hypertriglyceridaemia mainly in T2D patients [34] thereby FN can attenuate T2D‐induced AD by regulating lipid profile.

### 5.8 Long‐Chain Sphingolipids

Long‐chain sphingolipids are involved in the control of pancreatic β‐cell function and regulate insulin secretion [232]. Indeed, decreased long‐chain sphingolipid levels are linked with the progression of diabetic nephropathy [233]. An experimental study revealed that the ablation of very long‐chain sphingolipids induces the development of IR [234]. In AD, long‐chain sphingolipids are reduced and promote Aβ deposition and neuronal injury in AD [235, 236]. Long‐chain sphingolipids are diminished in AD in relation to the healthy controls [235, 236]; therefore, long‐chain sphingolipid could be a biomarker for AD neuropathology. In addition, sulfatide regulates pancreatic β‐cell function and neuronal activity. Therefore, the reduction of sulfatide occurs in the pathophysiology of AD and T2D [237, 238]. Of note, FN can reverse autoimmune response in nonobese diabetic mice by augmentation level of sulfatide and long‐chain sphingolipids in the pancreatic islet with subsequent improvement of pancreatic insulin sensitivity [48]. Different experimental studies revealed that sulfatide and long‐chain sphingolipids improve pancreatic β cells [49, 50]. Sulfatide and long‐chain sphingolipids are regarded as immune modulators and regulate insulin secretion from pancreatic β cells [51]. Moreover, FN protects against liver lipogenesis and hepatic IR by inhibiting the development of ER stress [52]. Consistently, sulfatide serum level is reduced in AD [237] and T2D [238]. These observations indicated that FN, through modulation of sulfatide and long‐chain sphingolipids, improves the pathogenesis of both T2DM and AD.

### 5.9 Kynurenic Acid

Research has demonstrated that the presence of mild inflammation and long‐term stress significantly raises the likelihood of prediabetes progressing into T2D [239]. It has been proposed that kynurenic acid, which is derived from tryptophan, is upregulated and stimulates the development of T2D by inducing the development of IR [239]. Therefore, kynurenic acid is augmented in T2D patients in relation to the controls. However, exercise regulates IR and energy expenditure through modulation levels of kynurenic acid [240]. It has been noted that the baseline level of kynurenic acid was increased in obese patients when they develop T2D [241], suggesting that a higher kynurenic acid serum level is correlated with T2D risk. Furthermore, kynurenic acid, which is produced from astrocytes, is also involved in AD neuropathology [242]. Increased brain kynurenic acid is regarded as a response to the inflammatory reactions in AD and other neurodegenerative ailments [242]. Notably, CSF kynurenic acid level is increased in AD cases in relation to healthy controls [242]. However, serum and erythrocytes kynurenic acid levels are reduced in AD due to an increase in kynurenine aminotransferase [243]. Remarkably, kynurenic acid is neuroprotective at low levels and neurotoxic at higher levels [244]; thus, it is regarded as a double‐sword edge in AD. Moreover, PPAR‐α agonist FN regulates the endogenous inhibitor of glutamate kynurenic acid [86]. PPAR‐α agonist gemfibrozil improves glutaminergic neurotransmission and associated memory function by inhibiting the synthesis of kynurenic acid [88, 89]. Therefore, PPAR‐α agonist FN improves both AD and T2D through the modulation of the synthesis and release of kynurenic acid.

### 5.10 PPAR‐γ Coactivator 1‐Alpha (PGC‐1α)

PGC‐1α is the master regulator of mitochondrial biogenesis by controlling gluconeogenesis, energy metabolism, upregulating of autophagy and the unfolded protein response [245]. PGC‐1α controls cholesterol homeostasis, blood pressure, insulin sensitivity and obesity [246]. Because PGC‐1α increases insulin sensitivity, there is a correlation between lower PGC‐1α expression and risk of IR [247]. A case–control research revealed a link between PGC‐1α gene polymorphism and the onset of T2D [248]. However, a prior investigation did not find a connection between T2D and PGC‐1α gene polymorphism [249].

Furthermore, PGC‐1α expression is highly reduced and negatively associated with Aβ accumulation and amyloid plaque in AD model [250]. Of interest, deficiency of PGC‐1α is linked with hyperactivity and behavioural changes in mice [251]. PGC‐1α has a neuroprotective role through anti‐inflammatory and antioxidant effects [252]. Moreover, PGC‐1α triggers the expression of neuroprotective receptors like PPAR‐α and estrogenic receptors [251, 252]. Suwa et al. [253], illustrated that the insulin‐sensitising drug metformin promotes the expression of PGC‐1α. Metformin has been revealed to be effective against AD neuropathology through the activation of PGC‐1α signalling [254]. In addition, PPAR‐α agonist promotes peripheral and central PGC‐1α expression [255]. Notably, FN, through activation of PGC‐1α signalling can reduce lipotoxicity in mice with diabetic nephropathy [256] and neuropathy [257]. Therefore, FN, through activation of PGC‐1α signalling, can have a dual role in mitigating T2D and AD neuropathology.

Taken together, FN has dual neuroprotective and antidiabetic effects that can mitigate AD neuropathology and T2D‐related complications through the modulation of various cellular processes and inflammatory signalling pathways. However, the present review has several limitations as the molecular and genetic mechanisms linking AD and T2D were not completely discussed. In addition, the reciprocal relationships between AD and T2D in relation to the effect of FN were focused on the immunoinflammatory relationship with little discussion on the metabolic pathway which connects AD and T2D. Despite these limitations, this review sheds light and gives a clue to the potential beneficial effect of FN against the development and progression of AD in T2D. To verify this concept, additional preclinical trials and prospective studies are recommended in this regard.

## 6 Conclusion

Evidence from epidemiological findings showed an association between T2D and AD. Due to the progression of brain IR and dysregulation of neuronal insulin receptors, T2D and IR enhance the risk of AD. These pathological modifications include aberrant deposition of Aβ plaques, reduced brain glucose metabolism, increased generation of Aβ_1‐42_ and impairment of Aβ clearance. Notably, PPAR‐α is highly dysregulated in T2D and AD. A PPAR‐α agonist FN which is mainly recommended in T2D patients with dyslipidaemia can improve lipid profile in T2D patients. Thus, FN is an effective agent in the attenuation of metabolic syndrome mediated by prediabetes. Similarly, FN improves impaired glucose tolerance through activation of PPAR‐α which inhibits BACE‐1, thereby reducing soluble APPβ and Aβ_42_ release and enhancing Aβ_1‐42_ clearance. In spite of the fact that Aβ is the primary pathology of AD; however, most clinical trials failed to confirm this hypothesis. Consequently, the intervention of early risk factors involved in AD neuropathology by PPAR‐α activators may reduce AD risk. Remarkably, PPAR‐α agonists induce a nonamyloidogenic pathway via activation of α‐secretase, which is also called ADAM10, leading to decreased AD neuropathology by shifting APP proteolysis towards nonamyloidogenic Aβ. Thus, PPAR‐α agonist FN seems to be effective in both AD and T2D; however, the underlying mechanistic role of FN in these conditions is not fully elucidated. It has been shown that FN via modulation of the immunoinflammatory signalling pathway can mitigate the pathogenesis of T2D and AD neuropathology. Furthermore, FN mitigates various cellular processes, including mitochondrial dysfunction, IR, oxidative stress, autophagy, immunoinflammatory disorders and ER stress, which are involved in the pathogenesis of T2D and AD neuropathology. Likewise, FN improves different mediators and signalling pathways that are intricate in the pathophysiology of both T2D and AD.

Taken together, FN has dual neuroprotective and antidiabetic effects that can mitigate AD neuropathology and T2D‐related complications through the modulation of various cellular processes and inflammatory signalling pathways.

## Author Contributions

**Mansour A. Alsaleem:** writing – review and editing (equal). **Hayder M. Al‐Kuraishy:** conceptualization (equal), conceptualization (equal), validation (equal), validation (equal). **Ali I. Al‐Gareeb:** resources (equal), validation (equal), visualization (equal). **Maha M. Abdel‐Fattah:** writing – review and editing (equal). **Mohammed Alrouji:** writing – review and editing (equal). **Nasser A. Al‐Harchan:** writing – review and editing (equal). **Mubarak Alruwaili:** writing – original draft (equal). **Marios Papadakis:** funding acquisition (lead), writing – original draft (equal). **Athanasios Alexiou:** supervision (equal), visualization (equal), writing – original draft (equal). **Gaber El‐Saber Batiha:** writing – review and editing (equal).

## Ethics Statement

The authors have nothing to report.

## Consent

The authors have nothing to report.

## Conflicts of Interest

The authors declare no conflicts of interest.

## Data Availability

All data are available in the manuscript.
